# Gene expression profiling in neuronal cells identifies a different type of transcriptome modulated by NF-Y

**DOI:** 10.1038/s41598-020-78682-8

**Published:** 2020-12-10

**Authors:** Tomoyuki Yamanaka, Haruko Miyazaki, Asako Tosaki, Sankar N. Maity, Tomomi Shimogori, Nobutaka Hattori, Nobuyuki Nukina

**Affiliations:** 1 Laboratory of Structural Neuropathology, Doshisha University Graduate School of Brain Science, 1-3 Tatara Miyakodani, Kyotanabe-shi, Kyoto, 610-0394 Japan; 2 Laboratory for Molecular Mechanisms of Brain Development, RIKEN Center for Brain Science, Saitama, 351-0198 Japan; 3 Department of Neuroscience for Neurodegenerative Disorders, Juntendo University Graduate School of Medicine, Tokyo, 113-8421 Japan; 4 Laboratory for Structural Neuropathology, RIKEN Brain Science Institute, Saitama, 351-0198 Japan; 5 Department of Genitourinary Medical Oncology, The University of Texas MD Anderson Cancer Center, Houston, TX 77030 USA; 6 Department of Neurology, Juntendo University Graduate School of Medicine, Tokyo, 113-8421 Japan

**Keywords:** Gene expression analysis, Sequencing, Cellular neuroscience, Transcription, Transcriptomics, Organelles

## Abstract

A heterotrimeric transcription factor NF-Y is crucial for cell-cycle progression in various types of cells. In contrast, studies using NF-YA knockout mice have unveiled its essential role in endoplasmic reticulum (ER) homeostasis in neuronal cells. However, whether NF-Y modulates a different transcriptome to mediate distinct cellular functions remains obscure. Here, we knocked down NF-Y in two types of neuronal cells, neuro2a neuroblastoma cells and mouse brain striatal cells, and performed gene expression profiling. We found that down-regulated genes preferentially contained NF-Y-binding motifs in their proximal promoters, and notably enriched genes related to ER functions rather than those for cell cycle. This contrasts with the profiling data of HeLa and embryonic stem cells in which distinct down-regulation of cell cycle-related genes was observed. Clustering analysis further identified several functional clusters where populations of the down-regulated genes were highly distinct. Further analyses using chromatin immunoprecipitation and RNA-seq data revealed that the transcriptomic difference was not correlated with DNA binding of NF-Y but with splicing of NF-YA. These data suggest that neuronal cells have a different type of transcriptome in which ER-related genes are dominantly modulated by NF-Y, and imply that NF-YA splicing alteration could be involved in this cell type-specific gene modulation.

## Introduction

NF-Y, also known as the CCAAT binding factor, is an evolutionarily conserved transcription factor composed of three subunits; NF-YA, NF-YB and NF-YC. It binds to CCAAT motif in the proximal promoter region to induce expression of various genes^1,2^. Despite its general binding consensus and ubiquitous expression, previous observations have highlighted the role of NF-Y in the cell-cycle progression of various types of cells, such as embryonic fibroblasts^3^ and tumor cell lines^4–6^. Furthermore, NF-Y is also critical for progression and maintenance of several stem/progenitor cells including hematopoietic stem cells (HSCs)^7–9^, myoblasts^10,11^ and embryonic stem (ES) cells^12–14^. Among these, in the HSCs and myoblasts, NF-YA expression is shown to be declined during differentiation^8,10^. In contrast, NF-YA splicing is altered during differentiation of ES cells; switching from short isoform lacking exon 3 (NF-YA-S)^15^ to long, full-length isoform (NF-YA-L)^12,13^. Finally, forced expression of NF-YA-S promotes the proliferation of these stem/progenitor cells^8,11^. Thus, one of the essential functions of NF-Y is the cell-cycle progression, in which NF-YA expression and/or splicing is critically involved.

On the contrary, recent studies using NF-YA knockout mice have suggested the role of NF-Y in endoplasmic reticulum (ER) homeostasis^16^. NF-YA remains to be expressed in differentiated brain neurons^17^, and its conditional knockout leads to progressive neurodegeneration accompanied by abnormal protein deposition in ER^18,19^. In addition, NF-YA knockout in differentiated hepatocytes induces liver degeneration and ER disorganization^20^. Direct regulation of ER-related genes by NF-Y is reported^18,21,22^. Thus, NF-Y is also critical for ER maintenance in some of the differentiated cells. However, there is no comprehensive gene-expression study for these cells, and whether NF-Y modulates a different transcriptome in these cells as well as the underlying mechanism regulating distinct cellular functions remains uncertain.

Here, we knocked down NF-Y in two types of neuronal cells, cultured neuro2a (N2a) cells and mouse brain striatal cells, and performed gene expression profiling. We found that the down-regulated genes preferentially contained NF-Y-binding motifs in their proximal promoters, and notably enriched genes related to ER functions rather than cell cycle-related genes. In contrast, profiling of HeLa and mouse ES cells revealed enrichment of cell cycle-related genes in down-regulated genes. Clustering analysis further identified several functional clusters where populations of down-regulated genes were highly distinct among the cell types. These data suggest that neuronal cells have a different NF-Y-transcriptome where ER-related genes are dominantly modulated. Further analyses using chromatin immunoprecipitation (ChIP) and RNA-seq data revealed that transcriptomic difference was not correlated with the DNA binding of NF-Y but partly with exon 3 inclusion of NF-YA, implying isoform-specific modulation of the transcriptome in neuronal cells.

## Results

### Gene expression profiling of NF-Y-knocked down N2a cells

**Figure 1:**
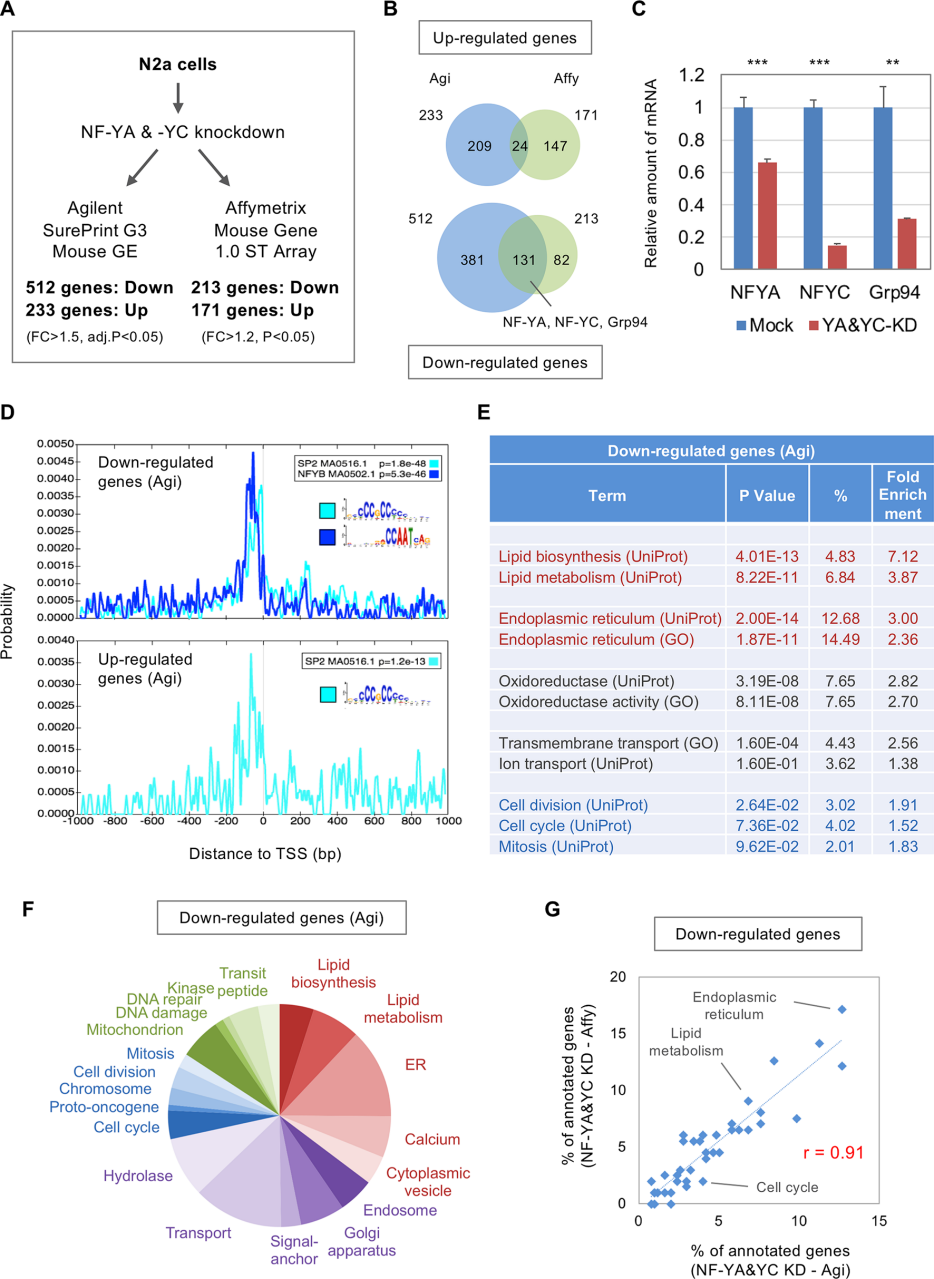
DNA microarray analysis of NF-Y knockdown N2a cells. (**A**) N2a cells were transfected with a knockdown vector for NF-YA and -YC or a control vector, and were processed for gene expression analysis using two DNA microarray systems. Analysis using Agilent arrays identified 745 DEGs of which 512 and 233 genes were down- or up-regulated, respectively, whereas analysis using Affymetrix arrays identified 384 DEGs of which 213 and 171 genes were down- or up-regulated, respectively. (**B**) Comparison of the DEGs identified by two DNA microarray analyses. Relative overlap was observed for down-regulated genes including NF-YA, NF-YC and Grp94. (**C**) qRT-PCR of NF-YA, -YC and Grp94 in control and knockdown cells. Values were means + s.d. of four data (***P < 0.001, **P < 0.01, t-test). (**D**) Genomic sequences of TSS ± 1000 bp for down- or up-regulated genes in Agilent arrays were analyzed by CentriMo to identify local enrichment of transcription factor binding motifs. In contrast to the SP2-binding CpG rich sequence, the NF-Y-binding CCAAT motif was specifically enriched in proximal region of TSS for down-regulated genes. (**E**) Gene-annotation enrichment analysis for down-regulated genes in Agilent arrays. *P* values, populations and fold enrichments were shown. Note the high enrichment of genes for lipid metabolism and ER (red) compared with those for cell cycle (blue). (**F**) Pie graph for the populations of annotated genes down-regulated in Agilent arrays. (**G**) Scatter plot for the populations of annotated genes down-regulated in Agilent or Affymetrix arrays. High correlation was observed on their populations (Pearson's correlation coefficient; r = 0.91).

To identify the transcriptome modulated by NF-Y in neuronal cells, we first knocked down NF-YA and -YC in a neuro2a (N2a) cell line, a mouse neuroblastoma cell line extensively used for various neurological and neuroscience studies^17,23–25^. DNA microarray analysis using Agilent DNA arrays identified 745 differentially expressed genes (DEGs) of which 512 were down-regulated including a known NF-Y-target, an ER chaperone Grp94 (Hsp90b1)^18^, in addition to NF-YA and -YC themselves (Fig. 1A,B, Supplementary Table S1). Their mRNA down-regulations were validated by quantitative reverse transcriptase-PCR (qRT-PCR) (Fig. 1C). The down-regulation of NF-YA and -YC proteins was further confirmed by western blot analysis (Supplementary Fig. S1), whereas reduction of Grp94 protein was not significant compared with that of mRNA, possibly due to a time-lag between reduced Grp94 transcription and the protein reduction.

We then picked up genomic sequences around transcription start sites (TSSs) of down- or up-regulated genes and screened transcription factor-binding motifs by a CentriMo software^26^ (Supplementary Fig. S2A). We observed an enrichment of NF-Y-binding CCAAT motifs in the regions proximal to TSSs for ~ 28% of down-regulated genes but not for up-regulated genes (Fig. 1D). In contrast, local enrichment of SP2-binding sequences was commonly observed for both up- and down-regulated genes (~ 30% of the genes) (Fig. 1D), which may be simply reflecting the CpG richness around TSSs. The proximal CCAAT motif-enrichment was not observed for genes picked up randomly or those dysregulated by knockdown of other transcription factors, USF1/2^27^, whereas USF1/2-binding E-box motifs were enriched in the latter case (Supplementary Fig. S2E,F). These data suggest that the CCAAT motif-enrichment is specific to the genes down-regulated by NF-Y knockdown in N2a cells, and imply that part of the gene down-regulations are direct consequences of reduced NF-Y-binding to their proximal promoters.

We then performed gene-annotation enrichment analysis for the down-regulated genes, and observed high enrichment for genes related to lipid metabolism/ER (red) compared with those related to cell cycle (blue) (Fig. 1E, Supplementary Table S2). Pie graph and scatter plot indicate that populations of the genes for lipid/ER/intercellular trafficking (red and purple) were higher than those for cell cycle/DNA damage/mitochondria (blue and green) (Fig. 1F, Supplementary Fig. S3). Thus, genes related to lipid/ER functions were preferentially down-regulated by NF-Y knockdown in N2a cells.

We further performed microarray analysis using Affymetrix DNA arrays (Fig. 1A), and in this case, low cut-off values were used because down-regulation of NF-YC and Grp94 was less efficient in the RNA samples used for the arrays (Supplementary Fig. S4A). Although obtained DEGs were lesser (384 DEGs; 213 down, 171 up) (Supplementary Table S3), a relative overlap was observed for the down-regulated genes with those identified by Agilent arrays (Fig. 1B). The proximal CCAAT motifs were confirmed to be enriched around TSSs of the identified down-regulated genes (Supplementary Fig. S2B). In addition, genes for lipid/ER/intercellular trafficking were highly populated in the down-regulated genes, even though higher cut-off value was used (Supplementary Fig. S4B–D, Supplementary Table S4). Finally, these gene-populations were highly correlated with those identified by Agilent arrays (Fig. 1G). Taken together, these data suggest that NF-Y dominantly modulates the transcriptome associated with lipid/ER functions in N2a cells.

**Figure 2:**
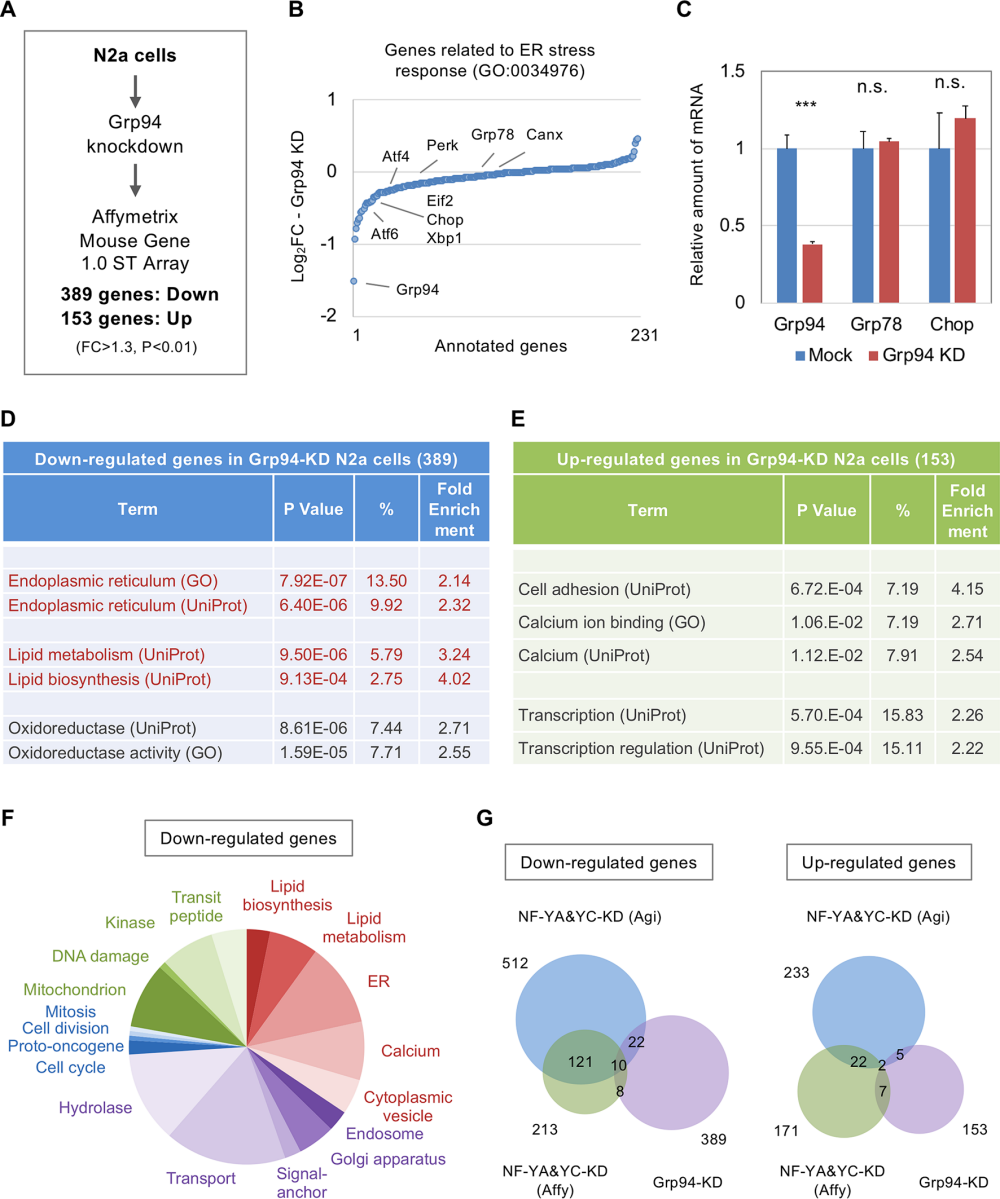
DNA microarray analysis of Grp94 knockdown N2a cells. (**A**) N2a cells were transfected with a knockdown vector for Grp94 or a control vector, and were processed for gene expression profiling using Affymetrix DNA microarray. Total 542 DEGs were identified, of which 389 and 153 genes were down- or up-regulated, respectively. (**B**) Log_2_FC values of the 231 genes annotated to ER stress response (GO 0034976). No distinct induction was observed for these genes. (**C**) qRT-PCR analysis of the ER stress-related genes. Values were means + s.d. of 4 data (***P < 0.001, t-test). (**D**,**E**) Gene-annotation enrichment analysis for down- (**D**) or up-regulated genes (**E**) by Grp94 knockdown. *P* values, populations and fold enrichments were shown. Relative enrichment was observed for genes related to ER and lipid metabolism (red) for down-regulated genes. (**F**) Pie graph for the populations of annotated genes down-regulated in Grp94-knockdown cells. (**G**) Comparison of the DEGs by Grp94 knockdown with those by NF-YA and -YC knockdown. Slight overlap was observed for down-regulated genes.

To examine the consequence of ER gene down-regulation by NF-Y knockdown, we then knocked down Grp94 in N2a cells and performed DNA microarray analysis. We identified 542 DEGs, of which 389 or 153 were down- or up-regulated, respectively (Fig. 2A, Supplementary Table S5). We observed down-regulation of Grp94 without up-regulation of ER stress-related genes such as Grp78 and Chop by DNA microarray, qRT-PCR and western blot analyses (Fig. 2B,C,E, S1), suggesting no induction of ER stress response by Grp94 knockdown. Genes related to lipid metabolism/ER were enriched whereas those related to cell cycle/ DNA damage were rarely observed (Fig. 2D,F, Supplementary Fig. S3, Supplementary Table S6). Because of slight overlaps of the DEGs among the Grp94- and NF-Y-knockdown cells (Fig. 2G), Grp94 down-regulation may be partially involved in the altered transcriptome mediated by NF-Y inactivation.

### Gene expression profiling of NF-Y-knocked down brain striatal cells

**Figure 3:**
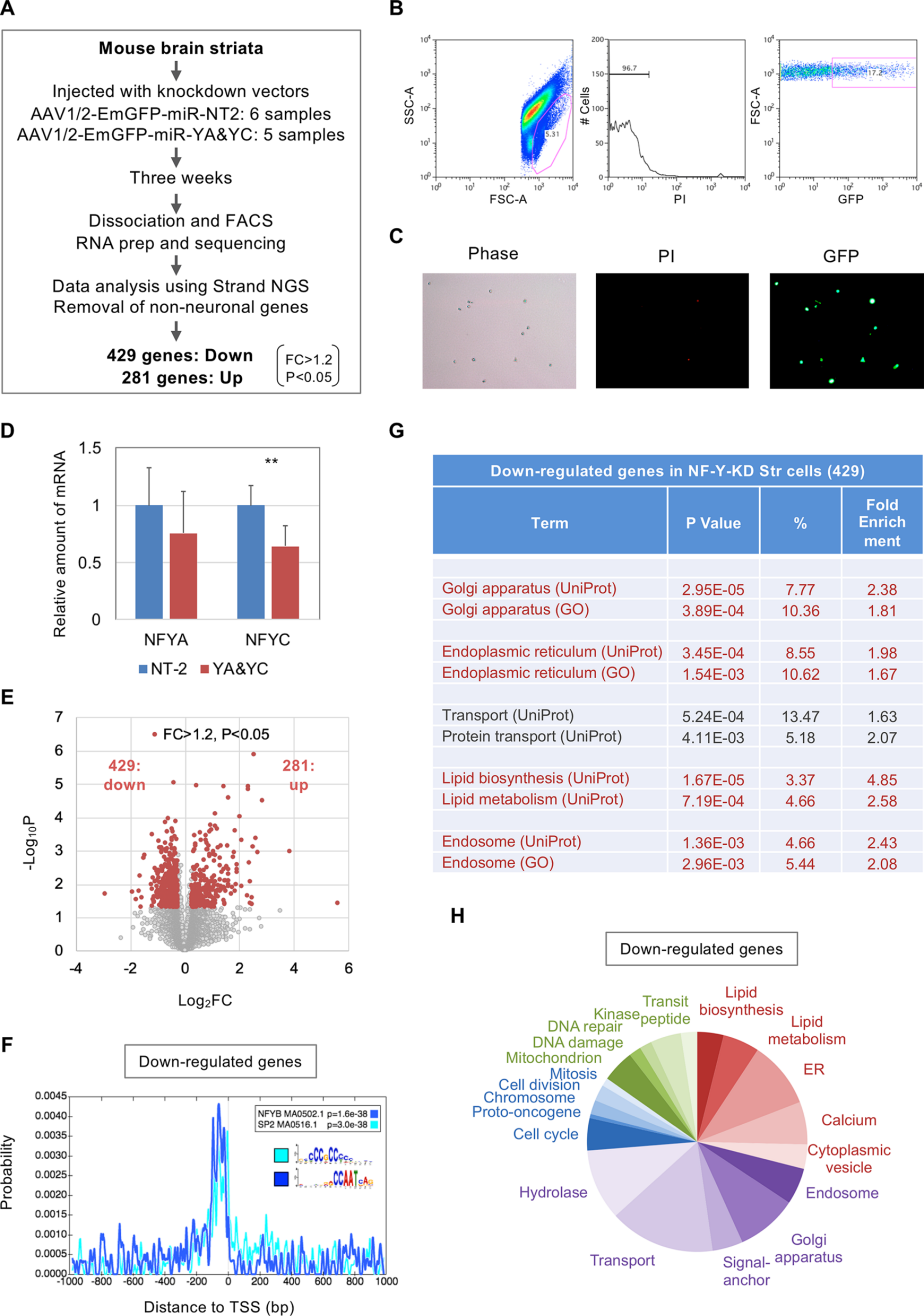
RNA seq analysis of NF-Y knockdown striatal cells in mouse brain. (**A**) Mouse brain striata were injected with the AAV1/2 vector encoding EmGFP-miR-NT2 (control) or that encoding EmGFP-miR-NF-YA and -YC (knockdown). After three weeks, striata were isolated and dissociated to sort EmGFP-positive cells by FACS. RNA was prepared and processed for RNA-seq analysis. After data analysis by Strand NGS software and removal of non-neuronal genes, total 710 DEGs were finally identified, of which 429 and 281 genes were down- or up-regulated, respectively. (**B**) Examples of FACS data. (**C**) Obtained EmGFP-positive and PI-negative cells after sorting. (**D**) qRT-PCR of NF-YA and -YC in control and knockdown striatal cells after sorting. Values were means + s.d. of 6 control and 5 knockdown samples (**P < 0.01, t-test). (**E**) Volcano plot of RNA-seq data. DEGs are colored by red. (**F**) CentriMo analysis of genomic sequences of TSS ± 1000 bp for the down-regulated genes. NF-Y-binding CCAAT motif was enriched in proximal regions in addition to the SP2-binding CpG rich sequence. (**G**) Gene-annotation enrichment analysis for down-regulated genes in NF-YA and -YC knockdown striatal cells. *P* values, populations and fold enrichments were shown. Relative enrichment was observed for genes related to Golgi, ER, lipid metabolism and endosome (red). (**H**) Pie graph for the populations of annotated genes down-regulated in NF-Y knockdown cells.

To identify the transcriptome modulated by NF-Y in in vivo neural tissues, we injected a virus vector for NF-Y knockdown (AAV1/2-EmGFP-miR-YA&YC) in mouse brain striata (Fig. 3A). A non-targeting vector (AAV1/2-EmGFP-NT2) was injected in different mice as controls. The mice were reared for three weeks after the injection. By immuno-staining of brain sections, we confirmed that EmGFP-expressing cells were positive for a neuronal marker NeuN (Supplementary Fig. S5). We then isolated striata and subjected them to fluorescence-activated cell sorter (FACS) to sort AAV-infected EmGFP-positive, but propidium iodide (PI)-negative living cells (Fig. 3B,C). After preparing total RNAs and checking their integrities (see method), the RNAs were then processed for cDNA synthesis and amplification. By qRT-PCR analysis, we confirmed reduced expression of NF-YC (Fig. 3D), whereas NF-YA reduction was not significant, possibly due to the relatively low effect of the knockdown construct (Fig. 1C).

We then performed RNA-seq analysis and identified ~ 1400 DEGs. However, genes expressing in glial cells were partly included, possibly due to partial infection of AAV1/2 to glial cells and/or contamination of glial cells during sorting. To remove these genes, we used a list of non-neuronally expressing genes in striatum, which was generated by analyzing our previous microarray data for isolated striatal neurons (see method)^28^. By subtracting these genes, we finally identified 710 DEGs of which 429 or 281 were down- or up-regulated, respectively (Fig. 3A,E, Supplementary Table S7). Again, local enrichment of NF-Y-binding motifs was observed only in the proximal promoters of down-regulated genes (Fig. 3F). Gene-annotation enrichment analysis revealed enrichment of the genes related to lipid metabolism/ER/Golgi rather than those for cell cycle/ DNA damage in the down-regulated genes (Fig. 3G,H, Supplementary Fig. S3, Supplementary Table S8), as observed in N2a cells. These data suggest that NF-Y modulates ER/lipid gene-dominant transcriptome also in brain striatal cells.

### Comparison of the transcriptomes modulated by NF-Y between neuronal and non-neuronal cells

**Figure 4:**
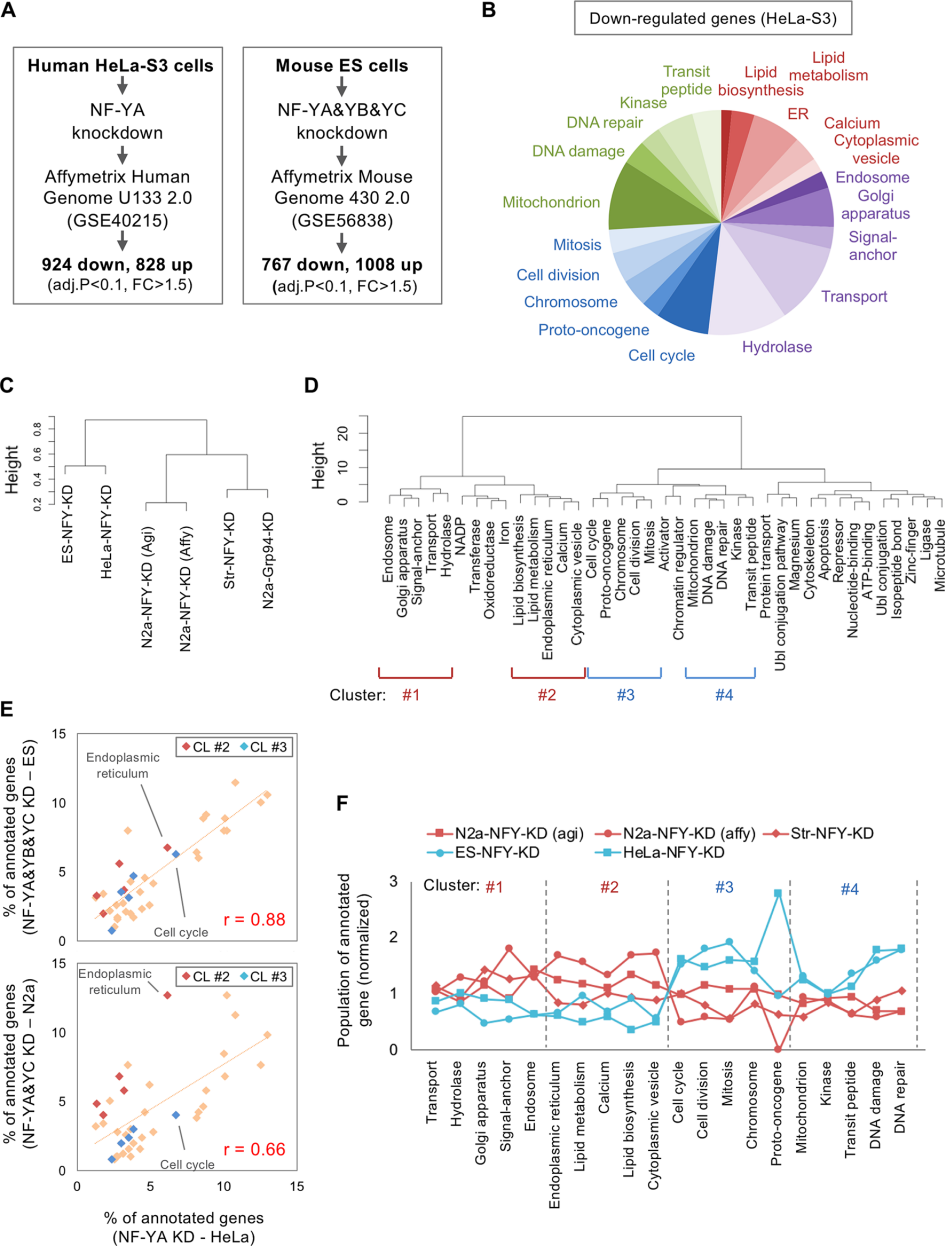
DNA microarray analysis of NF-Y knockdown HeLa and ES cells. (**A**) Analysis of DNA microarray data for HeLa cells with NF-YA knockdown or mouse ES cells with NF-YA, -YB and -YC triple knockdown identified 1752 (924 down, 828 up) or 1775 (767 down, 1008 up) DEGs, respectively. (**B**) Pie graph for the populations of annotated genes down-regulated in NF-YA knockdown HeLa cells. High enrichment was observed for cell cycle genes in these cells. (**C**,**D**) Hierarchical clustering of the analyzed cells based on the population of annotated genes (Ward.D2 method). (**C**) Clustering of the cells. HeLa and ES cells were in a same cluster and far from those of N2a/striatal cells. (**D**) Clustering of functional terms used for gene annotation. Clusters containing endosome/Golgi (CL #1), ER/lipid metabolism (CL #2), cell cycle/mitosis (CL #3) or mitochondrion/DNA damage (CL #4) were observed. (**E**) Scatter plot for the populations of annotated genes. The correlation was high between HeLa and ES cells (r = 0.88) whereas it was low between HeLa and N2a cells (r = 0.66) especially for those of CL #2 and #3. (**F**) Overall comparison of the populations of annotated genes in CL #1–4. Note that gene populations in CL #1 ~ 2 were relatively high in N2a and striatal cells whereas those for CL #3–4 were high in HeLa and ES cells.

To examine whether NF-Y modulates different transcriptomes in non-neuronal cells, we first analyzed microarray data for a human cervical carcinoma HeLa-S3 in which NF-YA was knocked down^29^ (Fig. 4A). Around 1000 down-regulated genes were identified (Fig. 4A, Supplementary Table S9), and interestingly genes for cell cycle/DNA damage/mitochondria were highly enriched (fold enrichment scores are 2.23, 1.70 and 1.89, respectively), compared with those for ER/lipid metabolism (1.24 and 1.42, respectively) (Fig. 4B, Supplementary Fig. S3, Supplementary Table S10). Their enrichment was also observed for 767 down-regulated genes in mouse ES cells with knockdown of NF-Y isoforms^14^ (Fig. 4A, Supplementary Fig. S3, Supplementary Tables S11, S12), and their gene populations were well correlated with those of HeLa cells (Fig. 4E). We confirmed local enrichment of NF-Y-binding motifs in proximal promoters of these down-regulated genes (Supplementary Fig. S1C,D), suggesting some of these genes are directly regulated by NF-Y.

Clustering analysis based on the gene populations also supports the similarity/difference among the cells, that is, HeLa and ES cells were in the same cluster and far from the clusters of N2a and striatal cells (Fig. 4C). Clustering focusing on functional term identified several clusters, among which we picked up four clusters (Fig. 4D); the one containing Golgi and endosome (#1), ER and lipid metabolism (#2), cell cycle and mitosis (#3), or DNA damage and mitochondria (#4). Populations of annotated genes, especially for CL #2 and #3 were highly diverse between HeLa and N2a cells, compared with those between HeLa and ES cells (Fig. 4E). Totally, gene populations for CL #1 ~ 2 were high in N2a and striatal cells whereas those for CL #3 ~ 4 were high in HeLa and ES cells (Fig. 4F).

Taken together, these data indicate that genes in different functional clusters were more significantly down-regulated in HeLa and ES cells, supporting the idea that NF-Y may modulate different transcriptomes between neuronal N2a/striatal cells and non-neuronal HeLa/ES cells.

### Comparison of the NF-Y-DNA binding between neuronal and non-neuronal cells

**Figure 5:**
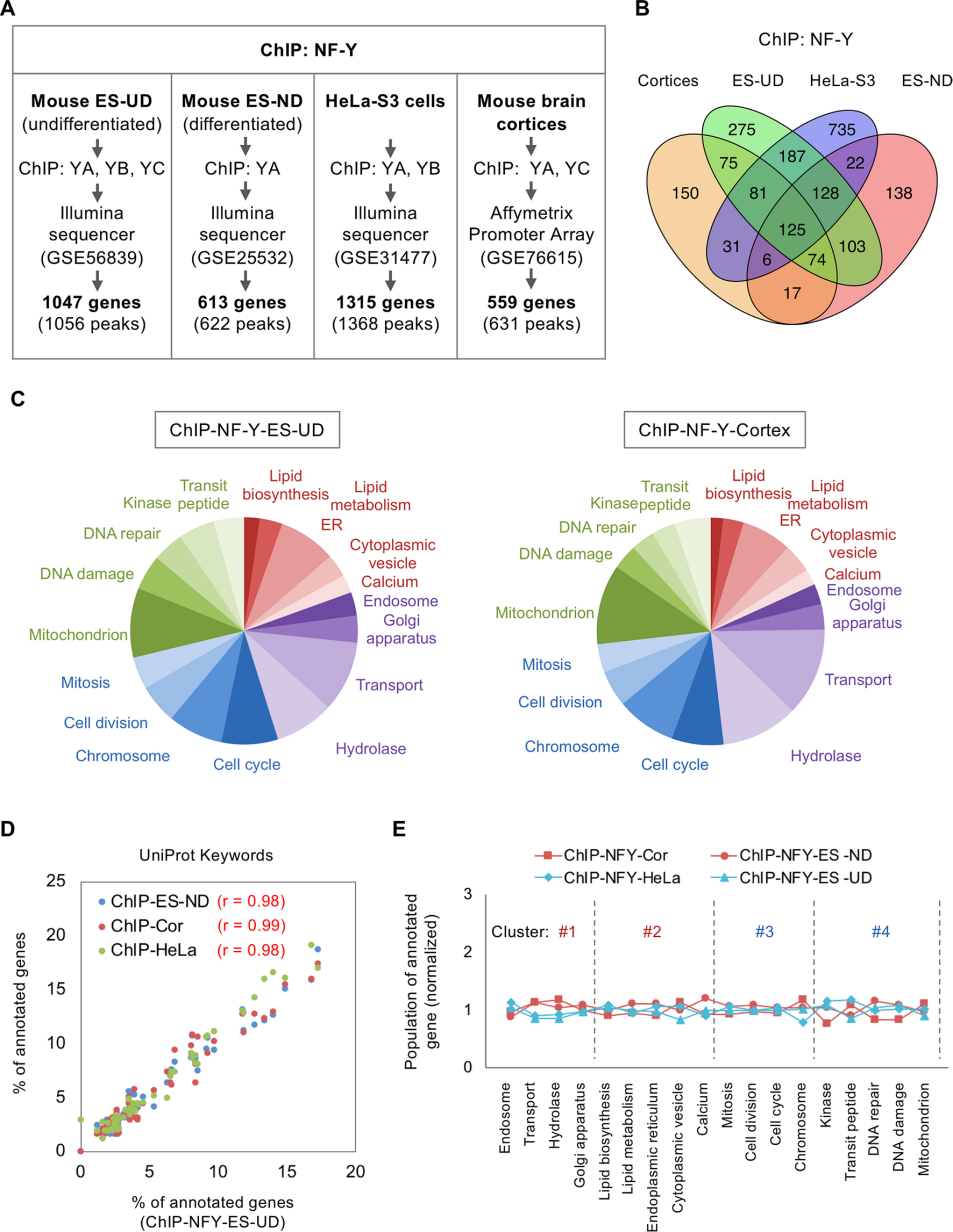
Analysis of NF-Y ChIP data in different types of cells. (**A**) ChIP data from mouse undifferentiated ES (ES-UD), nuerally differentiated ES (ES-ND), HeLa-S3 cells and mouse brain cortices were analyzed by a R package “ChIPpeakAnno”, and 600–1300 target genes of NF-Y were identified. (**B**) Comparison of the NF-Y target genes identified by ChIPs. Significant overlaps were observed even in different types of cells. (**C**) Pie graphs for the populations of annotated NF-Y-ChIP genes from ES-UD and brain cortices. They were mostly identical. (**D**) Scatter plot for the populations of annotated NF-Y-ChIP genes. Quite high correlations were observed among the four types of cells. (**E**) Overall comparison of the populations of annotated genes in CL #1–4. No distinct difference was observed in these clusters.

We next examined whether the difference in NF-Y-transcriptome is due to the difference in NF-Y-binding in these cells. For this purpose, we analyzed three sequence data for chromatin immunoprecipitation (ChIP-seq) of NF-Y from different types of cells; undifferentiated ES (ES-UD)^14^, neurally differentiated ES (ES-ND)^30^, and HeLa-S3 cells^31^; as well as our previous ChIP-chip data for mouse brain cortex^27^. We identified 600–1400 of potential NF-Y target genes that contain ChIP peak(s) within 2 Kbp to their TSSs (Fig. 5A, Supplementary Tables S13–S16). We confirmed that NF-Y-binding CCAAT motifs were significantly enriched in the proximal regions for ~ 50% of the target genes (Supplementary Fig. S6A–D). Notably, well overlaps were observed among the identified ChIP-genes (Fig. 5B). Furthermore, populations of annotated genes were mostly indistinguishable and closely correlated among the four ChIPs (Fig. 5C,D, Table Supplementary Table S17), even when focusing on functional clusters #1 ~ 4 (Fig. 5E). They were highly distinct from USF1/2-target genes identified by our previous ChIP-chip analysis (Supplementary Fig. S6E,F), suggesting the close correlations are specific to the NF-Y-target genes. This is in sharp contrast to the data of gene expression profiling (Fig. 4), thus suggesting the difference in NF-Y transcriptomes between neuronal and non-neuronal cells may not be due to the difference in NF-Y binding.

Because the NF-Y binding to distal promoters /enhancers and its potential role in cell type-specific transcription were reported^14,29^, we also examined NF-Y-target genes that contain ChIP peak(s) outside of 2 Kbp to their TSSs. However, the number of these genes was highly limited (~ 1/10 to the genes containing the peak(s) within 2 Kbp), and significant enrichment of genes related to ER/lipid metabolism/cell cycle/mitochondria was not observed (*P* > 0.1). These data suggest that at least for these genes, NF-Y binding to distal regions may not be mainly involved in their expressions.

### Comparison of NF-YA splicing between neuronal and non-neuronal cells

**Figure 6:**
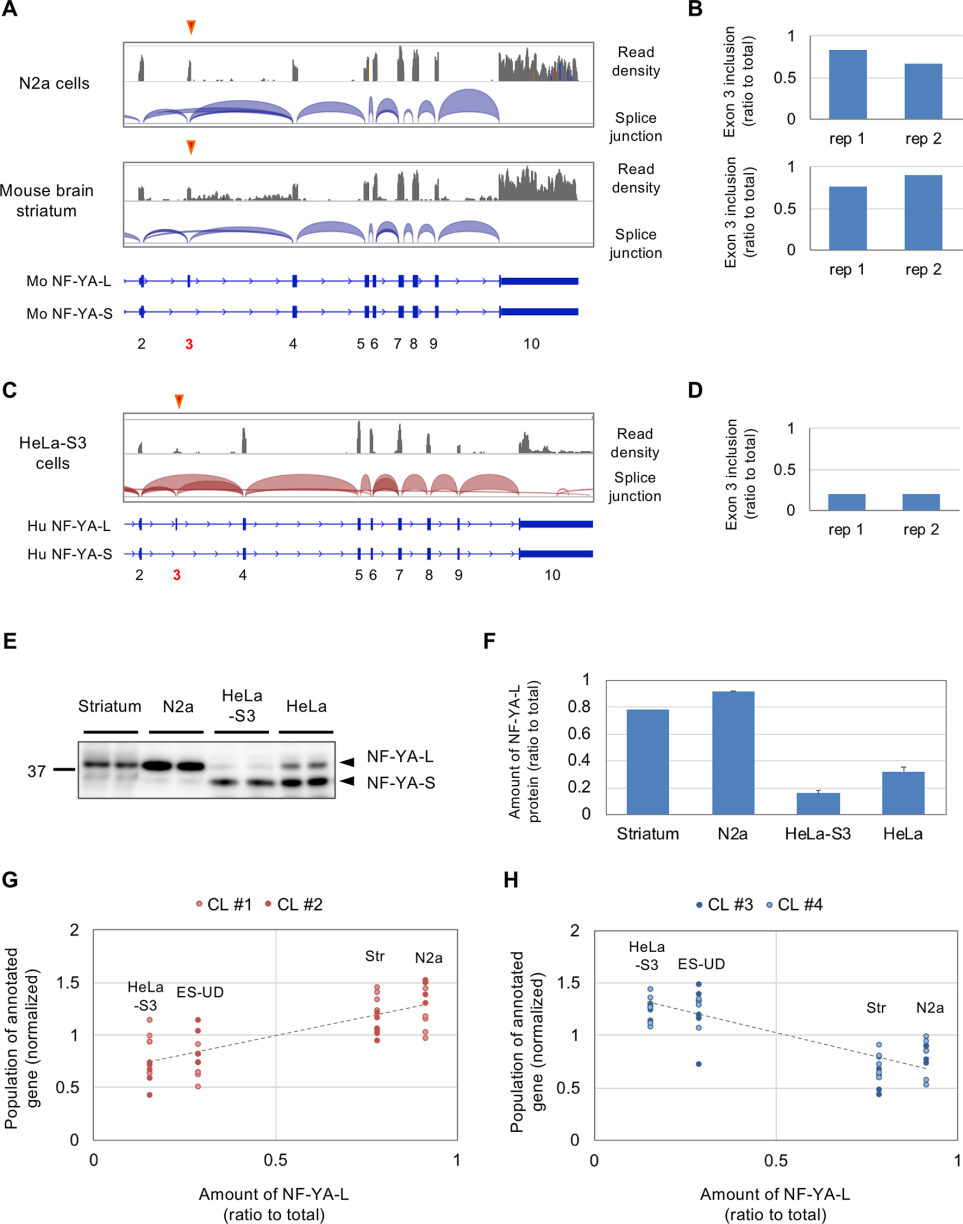
Analysis of NF-YA exon 3 splicing in different types of cells. (**A**) Integrative Genomics Viewer (IGV) shots of RNA-seq data showing read densities and splice junctions of NF-YA in mouse N2a cells and brain striatum. (**B**) Estimation of exon 3 inclusion in these cells/tissues (two replicates). (**C**) IGV shots of data for HeLa-S3 cells. (**D**) Estimation of exon 3 inclusion in HeLa-S3 cells (two replicates). (**E**) Striata isolated from B6 male mice, N2a cells, HeLa-S3 and HeLa cells were analyzed and subjected to western blotting using anti-NF-YA antibody. Bands for NF-YA-L and -S isoforms are indicated. (**F**) Ratios of NF-YA-L isoform to total NF-YA proteins were calculated by dividing the amount of NF-YA-L by sum of the NF-YA-L and -S proteins. Values are means of two (striatum) or three + s.d. data (N2a, HeLa-S3 and HeLa). (**G**,**H**) Comparison of the amounts of NF-YA-L isoform with gene populations in CL #1–2 (**G**) or CL #3–4 (**H**), showing partial positive or negative correlation, respectively.

Considering the dominant expression of NF-YA-S isoform and its critical role in cell cycle progression in ES cells^13^, we hypothesized that NF-YA isoform difference could be linked to the difference in NF-Y transcriptomes. To examine this, we first analyzed RNA-seq data for N2a cells and mouse brain tissues including striatum, cortex and hippocampus. We observed reads mapped to exon 3 of NF-YA to a similar extent as those to neighboring exons 2 and 4 (Fig. 6A, Supplementary Fig. S7A). By counting the reads spanning the exons, we estimated ratios of exon 3 inclusion in these cells/tissues as 0.7 ~ 1.0 (Fig. 6B, Supplementary Fig. S7B). In contrast, in HeLa-S3 cells, the density of the reads mapped to exon 3 was low and the ratio of exon 3 inclusion was estimated as ~ 0.2 (Fig. 6C,D, Supplementary Fig. S7C), indicating NF-YA-S is dominant in HeLa cells.

To confirm these, we performed western blotting and observed that NF-YA-L protein was dominant (~ 0.9 to total) in striatum and N2a cells (Fig. 6E,F), in consistent with our previous observations^17,18^. In contrast, NF-YA-S was dominant in HeLa-S3 and HeLa cell lines, and ratios of NF-YA-L to the total were 0.2 ~ 0.3 (Fig. 6E,F). The NF-YA-S dominance was also reported in mouse ES cells, and NF-YA-L ratio would be ~ 0.35 based on the blotting data^13^. We then compare the amount of NF-YA-L isoform with the populations of annotated genes in functional clusters. We observed a positive correlation for CL #1 and 2 but negative for CL #3 and 4 (Fig. 6G,H), suggesting a possible relationship between exon 3 inclusion/exclusion and NF-Y-transcriptome differences.

## Discussion

**Figure 7:**
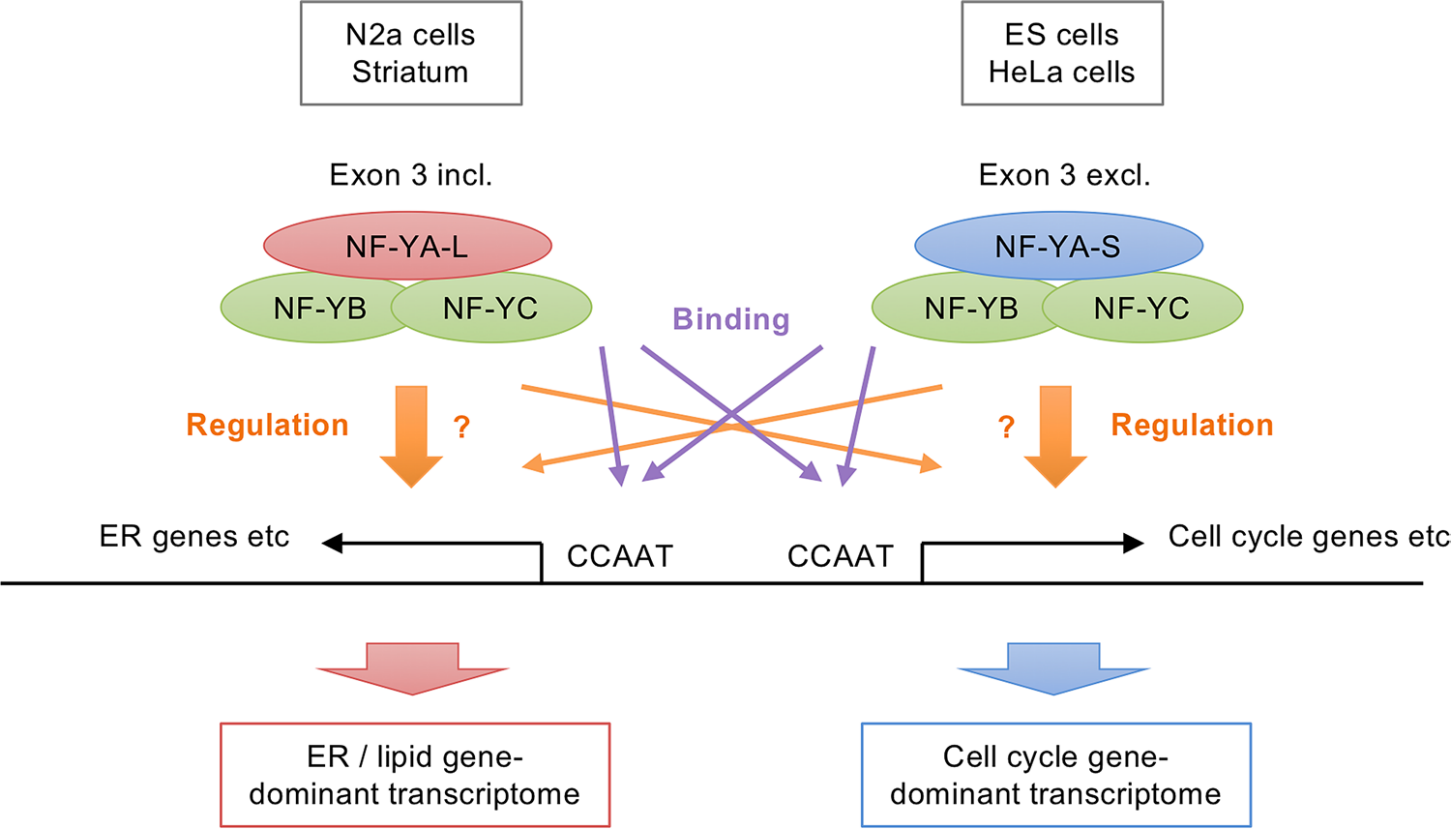
Schematic model of two distinct transcriptomes modulated by NF-Y complex containing different NF-YA splicing isoforms. Gene expression profiling in N2a and striatal cells suggests that neuronal cells contain the transcriptome in which ER/lipid genes were dominantly modulated by NF-Y. This contrasts with the NF-Y-transcriptome in HeLa and ES cells in which cell cycle/mitochondria genes were preferentially modulated. RNA-seq and ChIP data analyses suggest that NF-YA splicing alteration rather than DNA-binding preference could be involved in these cell type-specific transcriptomes.

By gene expression profiling of the NF-Y-knocked down N2a cells, we found preferential down-regulation of the genes related to ER/lipid metabolism. Similar transcriptomic alterations were also observed in NF-Y-knocked down brain striatal cells. These contrast with the profiling data of NF-Y-knocked down HeLa and ES cells in which distinct down-regulation was observed for genes related to cell cycle/mitochondria. Clustering analysis further identified several functional clusters where populations of the down-regulated genes were highly distinct. These data suggest that neuronal cells contain a different type of transcriptome where ER/lipid-related genes are dominantly modulated by NF-Y. Notably, ChIP analysis revealed close similarities in NF-Y-targeting to the genes between neuronal and non-neuronal cells, suggesting the transcriptomic difference is not explained by the difference in NF-Y targeting. Instead, we found a distinct difference in NF-YA splicing; exon 3 harboring NF-YA-L isoform is dominant in N2a and striatal cells whereas exon 3 excluding NF-YA-S isoform is dominant in HeLa and ES cells. Partial correlation was observed between the ratio of isoforms and the population of annotated genes. Thus, isoform difference could be involved in the cell type-specific gene modulation (Fig. 7).

NF-YA-S lacks exon 3 encoding 28 aa within the glutamine-rich transcriptional activation domain. Because it has similar activities to NF-YA-L isoform in DNA binding and gene transactivation^15^, the exon 3 splicing does not alter its basic transcription functions. However, it shows marked tissue-specific expression; NF-YA-S is dominant in thymus and spleen whereas NF-YA-L is dominant in the liver and brain^15^, as observed in this study. At the cellular level, NF-YA-S is predominant in stem/progenitor cells such as ES cells and HSCs whereas NF-YA-L is dominant in differentiated cells such as neurons. NF-YA-S is also highly expressed in several carcinoma cell lines derived from colon cancer (HCT116) and breast cancer (MCF7 etc.) and leukemia (K562)^11,13^. (Supplementary Fig. S7C) in addition to the HeLa cervical carcinoma cell lines. Notably, similarly to ES cells and HECs, knockdown of NF-YA-S in HCT116 cells induces cell proliferation defects^4^. Thus, the NF-Y complex containing NF-YA-S may be critical for cell proliferation. Studies using myoblasts further highlight the differential role of NF-YA isoforms; forced expression of NF-YA-S enhances myoblast proliferation whereas that of NF-YA-L boosts differentiation^11^, and the switch from NF-YA-L to -S impairs myoblast differentiation^32^.

Not only the differentiated cells, NF-YA-L is also predominant in some of proliferating cells including neuroblastoma cells (N2a shown here) and fibroblasts (NIH3T3, LMTK, NHLF, etc.) (Supplementary Fig. S7C)^15^. We found that even in proliferating N2a cells, ER/lipid-related genes were dominantly down-regulated by NF-Y knockdown. We thus speculate that difference in NF-YA isoform rather than cell proliferation propensity determines the type of transcriptome in which either ER/lipid-related genes or cell cycle-related genes were preferentially modulated by NF-Y (Fig. 7). However, its underlying mechanism is so far uncertain. One possibility is isoform-specific networks of protein–protein interactions that mediate differential gene transcriptions. Further analysis is necessary to clarify this point.

In summary, our gene expression profiling in neuronal cells identified a distinct transcriptome where ER/lipid-related genes are dominantly modulated by NF-Y containing NF-YA-L isoform. Further transcriptomic studies in different cellular contexts^2,33^ may deepen our understanding of how ubiquitous transcription factors such as NF-Y could modulate different transcriptomes to mediate distinct cellular functions.

## Methods

### Cell culture and transfection

Mouse neuro2a (N2a) neuroblastoma cells and human cervical carcinoma cells, HeLa-S3 and HeLa, were maintained in DMED supplemented with 10% fetal bovine serum and penicillin–streptomycin in an atmosphere containing 5% CO_2_. For knockdown, we used miR RNAi expression vectors for NF-YA and -YC (YA-8/YC-2) or Grp94 (Grp94-1, -5) in pcDNA6.2-GW/EmGFP-miR vector (Invitrogen)^18^. An empty vector and a vector expressing non-targeting miR RNAi (NT2) were used as controls^18^. Cells were transfected with these RNAi expression vectors using Lipofectamine 2000 regions (Invitrogen), and incubated further two days to induce efficient gene knockdown.

### Quantitative RT-PCR and DNA microarray

Total RNAs were prepared from transfected neuro2a cells by Qiagen RNeasy kit according to the manufacture's protocol. For quantitative RT-PCR (qRT-PCR), obtained RNAs were subjected to reverse transcription (RT) using a ThermoScript RT-PCR System (Invitrogen) to synthesize cDNAs. The cDNAs were mixed with primers and FastStart Universal SYBR Green Master Mix (Roche, Basel, Switzerland) and subjected to qPCR using a LightCycler 480 II system (Roche). Primers for qPCR were described previously^18^. All values obtained were normalized with respect to those of GAPDH. For DNA microarray, we used two array systems obtained from Agilent and Affymetrix. As for Agilent system, we prepared labeled cDNAs from total RNA by an Agilent Low Input Quick Amp Labeling Kit One-Color, and hybridized them to Agilent SurePrint G3 Mouse GE 8 × 60 K Microarray according to the manufacture's protocols. As for Affymetrix system, we prepared labeled cDNAs by Ambion WT Expression Kit and Affymetrix WT Terminal Labeling Kit, and hybridized them to Affymetrix Mouse Gene ST arrays according to the manufacture's protocols. Obtained microarray data were analyzed using an Agilent GeneSpring GX software to identify differentially expressed genes (DEGs) by gene knockdown. Genes with low expressions (less than 20th percentile) were excluded to reduce false positives. The analyzed data are listed in Supplementary Table S18.

### Western blotting

SDS-PAGE and western blotting were performed as described previously^34^. Following primary antibodies were used for detection; anti-NF-YA (YA1-5AP) generated against conserved C-terminal 14 a.a. peptide of NF-YA that reacts with both NF-YA-L and -S isoforms^17^; anti-NF-YC (YC5-3AP) generated against its C-terminus^17^; anti-Grp94 purchased from Enzo (SPA-850); anti-Grp78 from BD (610978); and anti-beta-actin from Sigma (A5441). Chemiluminescent signals were obtained and quantified using an ImageQuant LAS-4000 (GE). Full-length images of Western blots are shown Supplementary Fig. S8.

### AAV vector injection and cell sorting

The mouse experiments were approved by the animal experiment committees at RIKEN and Doshisha University. Mice were maintained and bred in accordance with guidelines of RIKEN and Doshisha University. All methods were performed in accordance with the guidelines and regulations of RIKEN and Doshisha University. The adeno-associated virus (AAV) vectors expressing EmGFP with miR RNAi for NF-YA and -YC (YA-8/YC-2) or control non-targeting miR RNAi (NT2) under CAG promoter were prepared by GeneDetect. The AAV vectors were stereotaxically injected into striatum in both hemispheres of 6-week-old wild-type male B6 mice as described previously^19^. Three weeks after the injection, the mice were deeply anesthetized by peritoneal injection of tribromoethanol (Avertin). Part of the mice were subjected to immunofluorescence analysis^19^ to check AAV infection in striatal neurons by using an antibody against NeuN (Chemicon MAB377). From the remaining mice, striata were surgically isolated and dissociated by Papain Dissociation System (Worthington Biochemical Corporation) according to the manufacture's protocol. To isolate RNAi-expressing cells positive for green fluorescence (EmGFP), dissociated cell suspensions were stained with 20 μg/mL of propidium iodide (PI) (Sigma) and then sorted by FACS Aria (BD Biosciences) as described previously^28^. After sorting, 27,000–100,000 EmGFP-positive cells without PI stain were obtained.

### cDNA preparation and RNA-seq for sorted striatal cells

Total RNAs were prepared from the sorted cells by RNeasy Micro Kit (Qiagen), and analyzed by an Agilent Bionalyzer. Their RNA integrity numbers (RINs) were 6.7–9.0 (average is 8.2) and obvious RNA degradation was not observed. Because the RNA concentrations were low (~ 2 ng/μL), we used a Clontech SMARTer Ultra Low Input RNA Kit for Sequencing (v3) for efficient amplification of cDNA after reverse transcription according to the manufacture's protocol. Libraries for sequencing were prepared by a Nextera XT Sample Prep Kit, and sequenced by an Illumina HiSeq 2500 system (single read; 50 cycles; rapid run mode). Total 11 samples were analyzed in two sequencing lanes and finally 24–37 M reads (mean qualities > 37) were obtained for each sample. Obtained seq data were analyzed using Agilent Strand NGS to identify DEGs by NF-Y knockdown. Genes with lower expressions (max of the read counts is less than 200) were excluded. We also excluded genes dominantly expressed in non-neuronal cells in striata. The list of these genes was generated from our previous microarray data^28^, that is, the data for isolated striatal neurons were compared with those for total striatum to obtain the non-neuronal genes whose expressions were decreased to less than -fivefold in isolated striatal neurons. The analyzed data are listed in Supplementary Table S18.

### Analysis of DNA microarray data

For DNA microarray data for NF-Y-knockdown mouse ES cells (GSE56838)^14^ and HeLa-S3 cells (GSE40215)^29^, those were analyzed by GEO2R in GEO website to identify DEGs. We also used our previous DNA microarray data; gene expression array for USF1/2-knockdown N2a cells (Affymetrix Mouse Gene ST arrays; GSE76615) and NF-Y-ChIP-chip and USF-ChIP-chip from mouse brain cortex (Affymetrix Mouse Promoter 1.0R Array; GSE76616)^18,27^. The ChIP-chip data were re-analyzed by a R package ChIPpeakAnno^35^ after converting our previous data to mm10-based BDE format files by LiftOver in UCSC Genome Browser. The data used for analysis are listed in Supplementary Table S19.

### Analysis of RNA-seq data

For RNA-seq data of N2a cells (GSE106999), mouse brain striata and cortices (GSE103715)^36^, mouse brain hippocampi (GSE83931)^37^, HeLa-S3 cells (GSE90235)^31^, Sequence Read Archive (SRA) data were retrieved from GEO/SRA database and extracted by SRAToolkit. After quality check by FastQC, the reads were subjected to quality control by Trimmomatic^38^ and PRINSEQ^39^. Trimmed reads were then aligned to mouse (mm10) or human (hg38) genomes by HISAT2^40^, and obtained SAM format files were converted to BAM format files by Samtools^41^. Read densities and splice junctions were visualized using Integrative Genomics Viewer (IGV)^42^. To examine exon 3 inclusion, reads spanning exons 2–3, 3–4 or 2–4 were manually counted, and sum of the former twos was divided by sum of total counts where counts for 2–4 were doubled. RNA-seq data for various human cell lines were from ENCODE/Caltech (GSE33480)^43^ and visualized with GEO genome data viewer. The data used for analysis are listed in Supplementary Table S19.

### Analysis of ChIP-seq data

For NF-Y-ChIP-seq data of undifferentiated mouse ES cells (GSE56839)^14^, neurally differentiated ES cells (GSE25532)^30^ and normal HeLa-S3 cells (GSE31477)^31^, SRA data were retrieved and quality-checked as above. The reads were subjected to quality control by Trimmomatic, followed by aligned to mouse (mm10) or human (hg38) genomes by Bowtie 2^44^. After converting to BAM format files, NF-Y-ChIP peaks were called by MACS2^45^ where ChIP-inputs were used as controls. Obtained peak files (BED format) were analyzed by a R package ChIPpeakAnno to obtain overlapping peaks (max gap = 100 bp) among the ChIPs using antibodies against different NF-Y isoforms. Then, the peaks were gene-annotated using a R package EnsDb.Mmusculus.v79. We selected the peaks locating within ± 2000 bp of the TSS as gene-annotated peaks. The data used for analysis are listed in Supplementary Table S19.

### Bioinformatics

Gene-annotation enrichment analysis was performed using DAVID bioinformatics database^46^. *P* values, populations and fold enrichments of functional terms were obtained. Venn diagrams were drawn by a R package VennDiagram^47^ or a web application BioVenn^48^. Clustering was performed by a R package hclust with Ward's method. Correlation coefficients were calculated by a R package psych. To identify locally enriched TF binding motifs around TSS, we first created a BED file containing information of TSS+/− 1000 positions for all genes. After extracting the information for selected genes, we then created a fasta file containing genomic sequences of TSS+/− 1000 for selected genes by ChIPpeakAnno, followed by analysis with a web application CentriMo to identify locally enriched transcription factor binding motifs^26^.

### Database

All of the original data for DNA microarray and RNA-seq have been deposited in GEO database under accession numbers GSE151144, GSE151145, GSE151146 and GSE151275.

## Supplementary information

Supplementary Figures.

Supplementary Tables.
