# Supplementary material for: Gene expression profiling in neuronal cells identifies a different type of transcriptome modulated by NF-Y
Source: Sci Rep. 2020 Dec 10;10:21714. doi: 10.1038/s41598-020-78682-8 (PMC7728767; doi:10.1038/s41598-020-78682-8)
Supplement: Supplementary file 1 — Supplementary Figures. [file 41598_2020_78682_MOESM1_ESM.pdf]

Gene Expression Profiling in Neuronal Cells Identifies a Different Type of Transcriptome Modulated by NF-Y

Tomoyuki Yamanaka, Haruko Miyazaki, Asako Tosaki, Sankar N. Maity, Tomomi Shimogori, Nobutaka Hattori and Nobuyuki Nukina

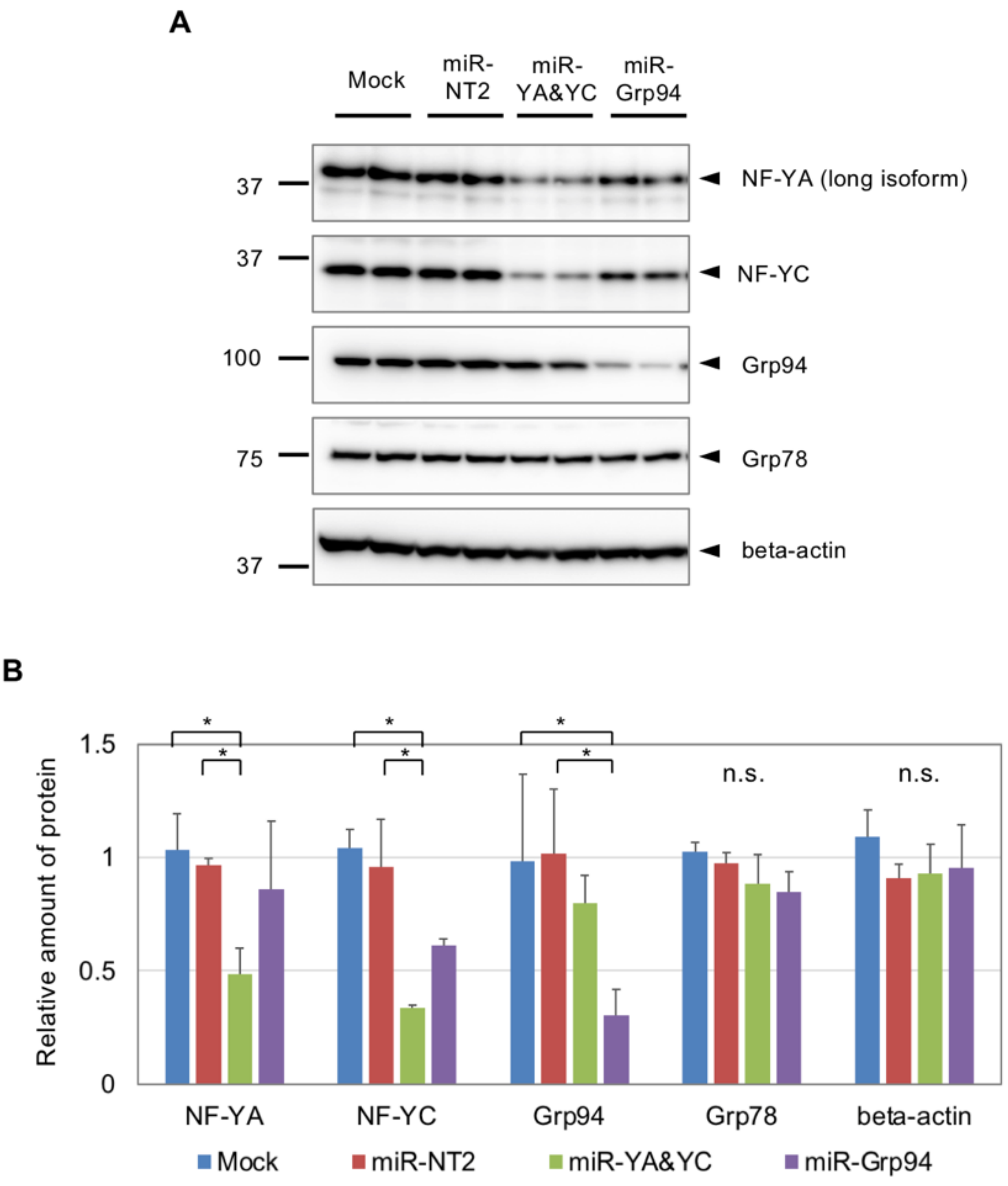

Supplementary Figure S1. Western blot analysis of N2a cells transfected with knockdown vectors.

(A) N2a cells were transfected with EmGFP-miR vector (mock) or that for non-targeting control (NT2), NF-YA and -YC (YA&YC) or Grp94. Two days later, the cells were subjected to western blotting using antibodies for NF-YA, NF-YC, Grp94, Grp78 and beta-actin. (B) Quantification of the proteins. Values are means + s.d. of three data, and statistically analyzed by one-way ANOVA followed by Tukey post-test (*significant, n.s.; not significant). Note that significant down-regulation of NF-YA (L isoform), NF-YC and Grp94 protein was observed by respective miR constructs, whereas Grp78 expression was not altered by these constructs.

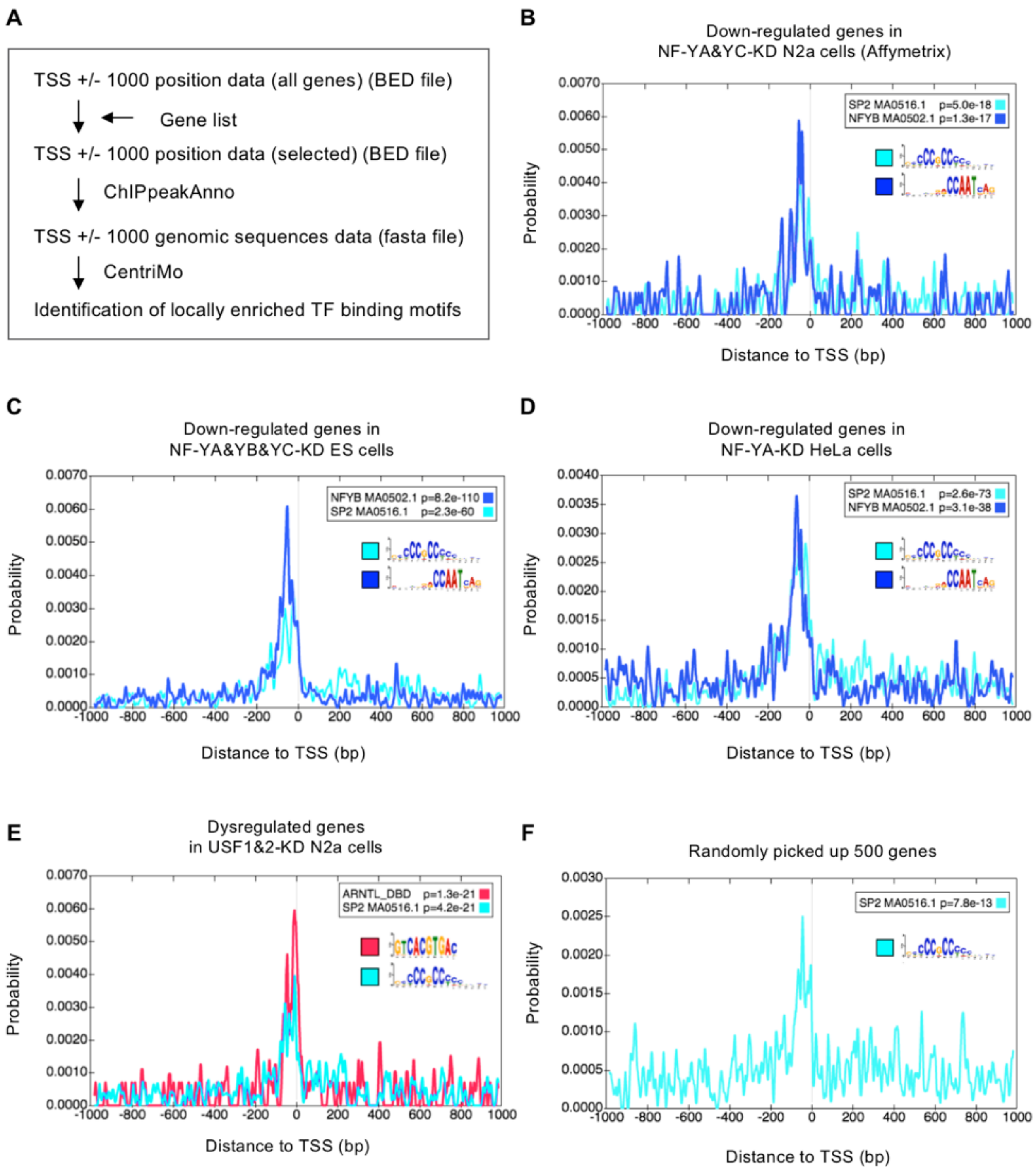

Supplementary Figure S2. Analysis of local enrichment of transcription factor-binding motifs around TSSs of down-regulated genes.

(A) Scheme of motif enrichment analysis around TSSs by CentriMo software. (B-D) Motif analysis of down-regulated genes in NF-YA and -YC knockdown N2a cells (Affymetrix array) (B), those in NF-YA, -YB and -YC knockdown ES cells (C), and those in NF-YA knockdown HeLa cells (D). (E) Motif analysis of dysregulated genes in USF1/2-knockdown N2a cells. (F) Motif analysis of randomly picked up mouse genes.

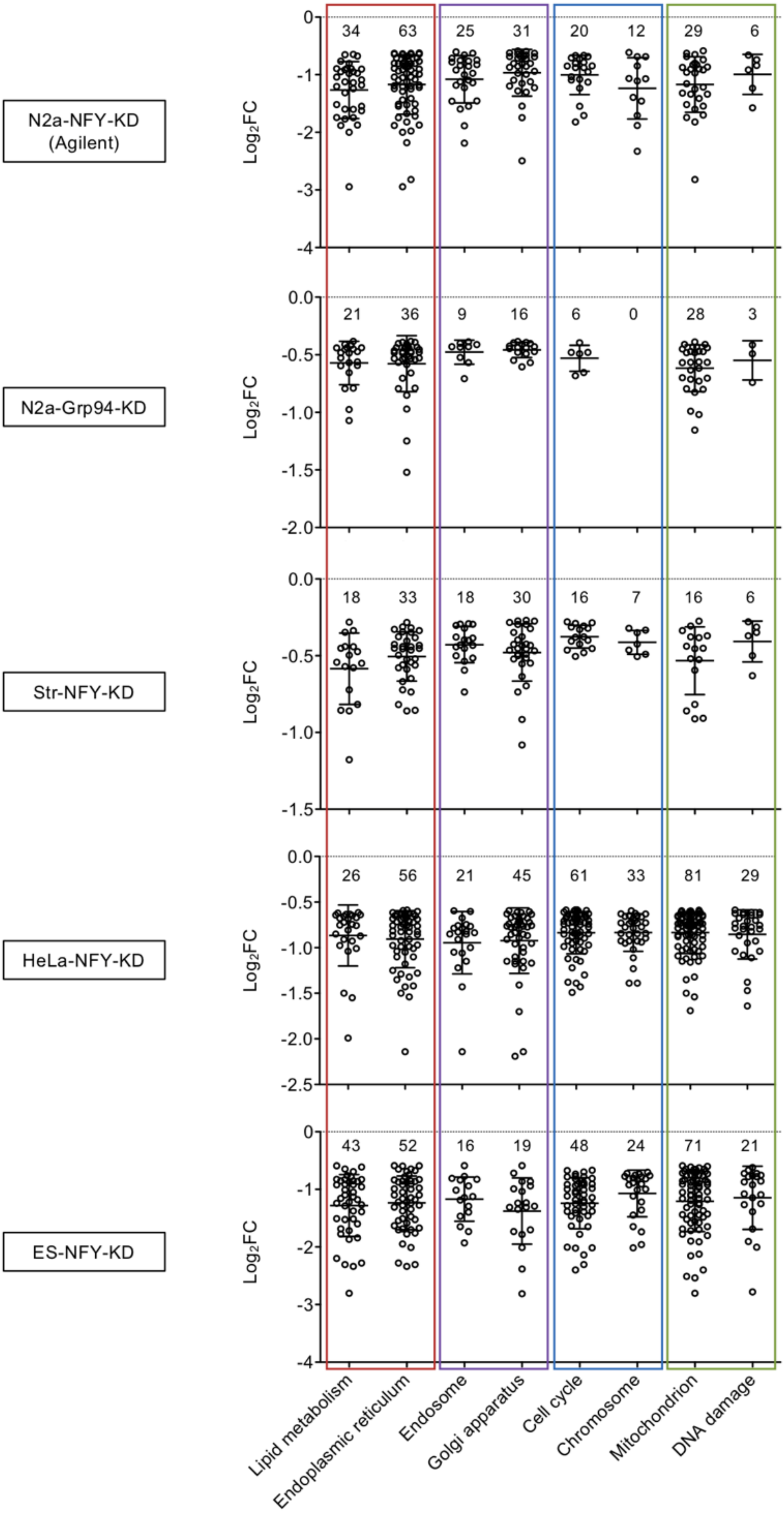
N2a-NFY-KD (Agilent)
N2a-Grp94-KD
Str-NFY-KD
HeLa-NFY-KD
ES-NFY-KD
Log2FC
34
63
25
31
20
12
29
6
21
36
9
16
6
0
28
3
18
33
18
30
16
7
16
6
26
56
21
45
61
33
81
29
43
52
16
19
48
24
71
21
Lipid metabolism
Endoplasmic reticulum
Endosome
Golgi apparatus
Cell cycle
Chromosome
Mitochondrion
DNA damage

Supplementary Figure S3. Scatter plots of down-regulated genes in different functional categories.

Identified down-regulated genes by NF-Y or Grp94 knockdown in indicated cells were categorized based on their functional annotations and plotted. Numbers of the down-regulated genes are indicated. The genes in left two categories were abundant in N2a and striatal cells whereas genes in right two became obvious in HeLa-S3 and ES cells.

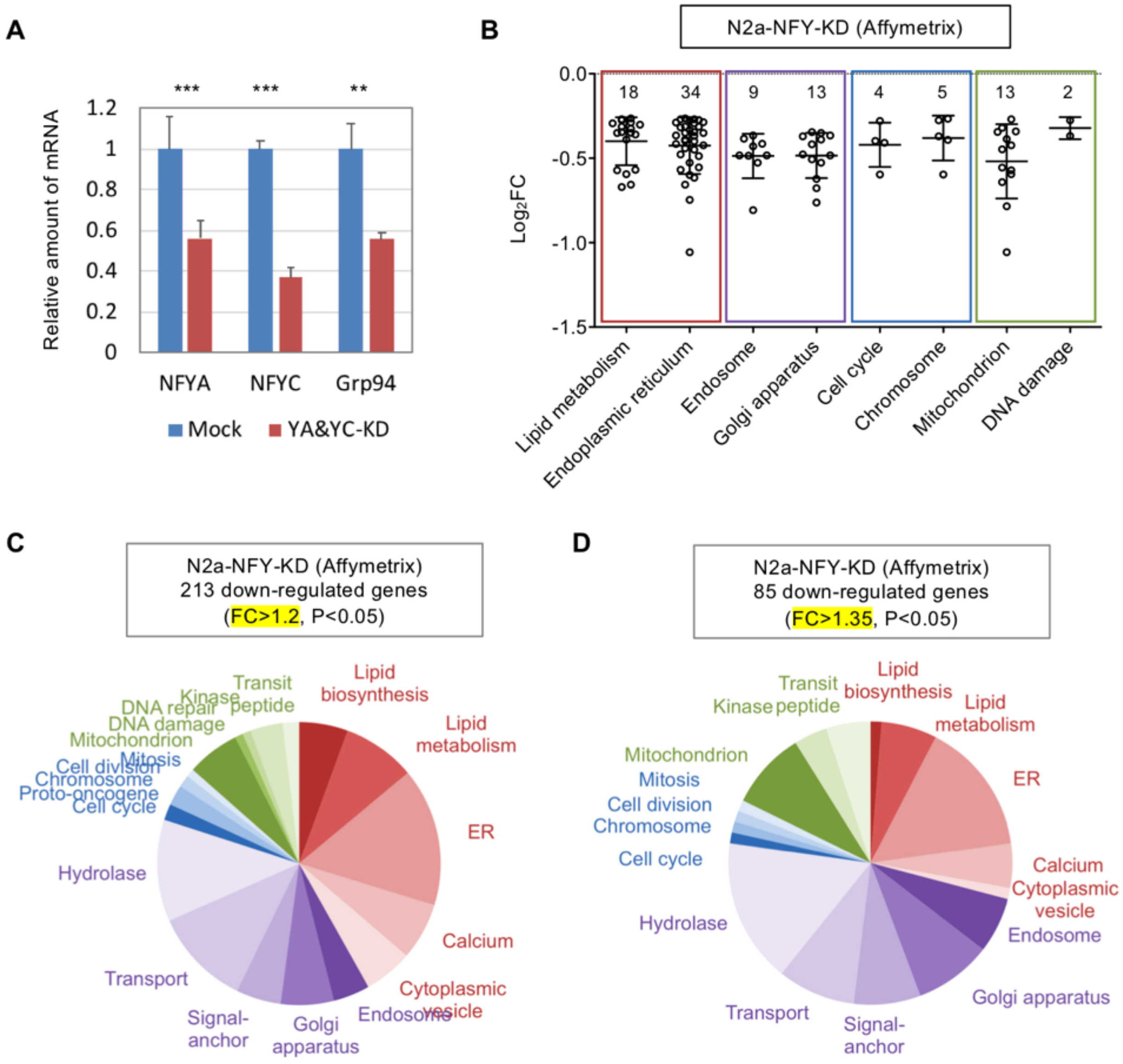

Supplementary Figure S4. Analysis of Affymetrix microarray data for N2a cells with NF-YA and -YC knockdown.

(A) qRT-PCR of NF-YA, -YC and Grp94 from RNA samples used for Affymetrix microarray analysis. Values were means +s.d. of three data (***P < 0.001, **P < 0.01, t-test). Although their significant down-regulation was observed, down-regulation of NF-YC and Grp94 was less efficient compared with the RNA samples used for Agilent microarray analysis (Figure 1C). (B) Scatter plot of down-regulated genes in different functional categories. Numbers of the down-regulated genes are indicated. The genes in left two categories were abundant. (C) Pie graph for the populations of annotated genes down-regulated in Affymetrix array data at low (FC>1.2) (C) or relatively high threshold (FC>1.35) (D). Although identified genes were drastically decreased by high threshold cut-off, ER-related genes were still abundant whereas those related to cell cycle were less abundant.

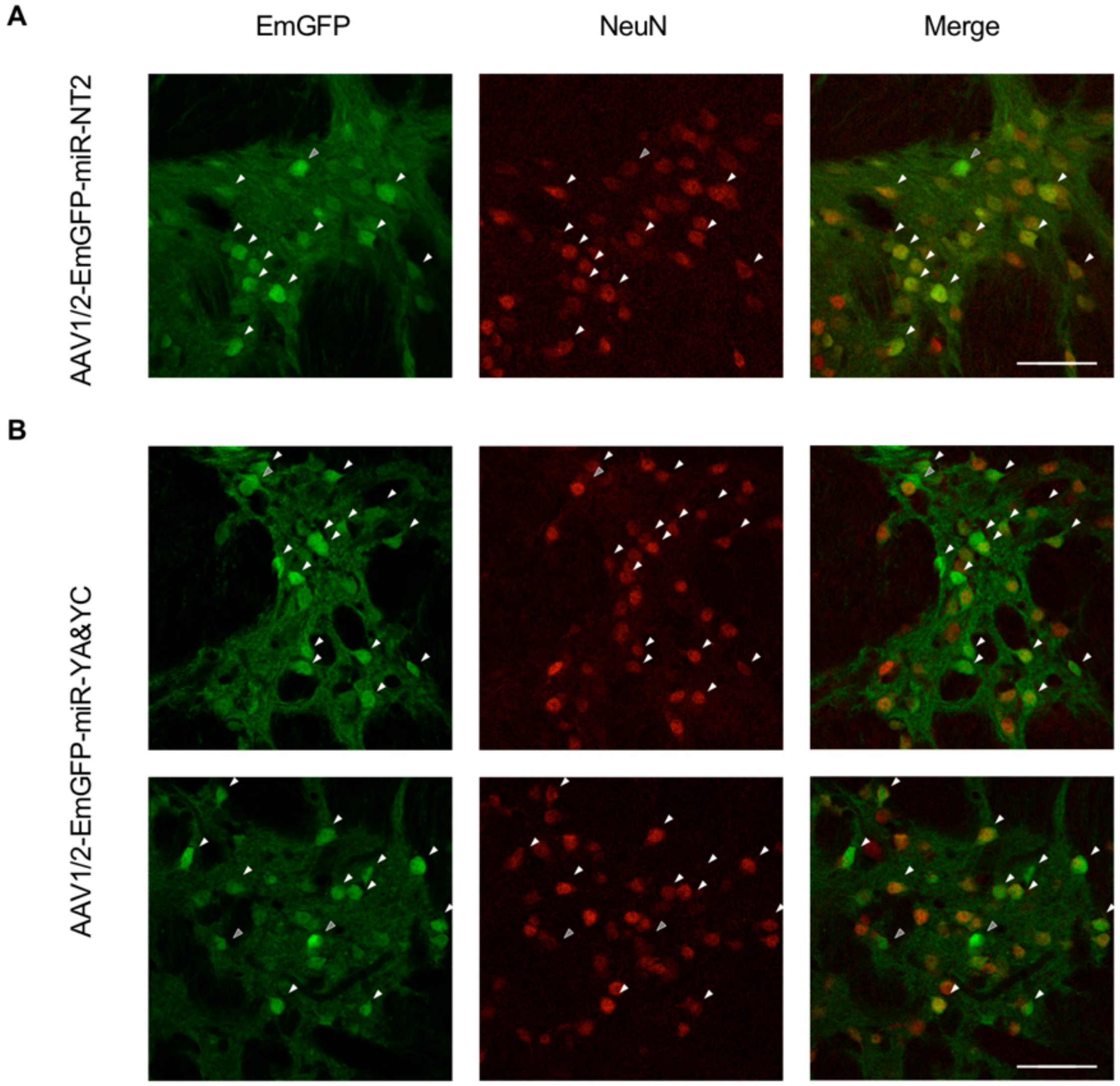

Supplementary Figure S5. Immunofluorescence analysis of brain sections for AAV-injected mice.

Mouse brain striata were injected with the AAV1/2 vector encoding EmGFP-miR-NT2 (control) (A) or that encoding EmGFP-miR-YA&YC (knockdown of NF-YA and -YC) (B). After three weeks, mice were fixed and striatal sections were stained with anti-NeuN antibody. In either infection, most of EmGFP-expressing cells were positive for NeuN (white arrowheads) while a few of them were negative for it (gray arrowheads). Data of two biological replicates were shown for B. Scale bars are 50 μm.

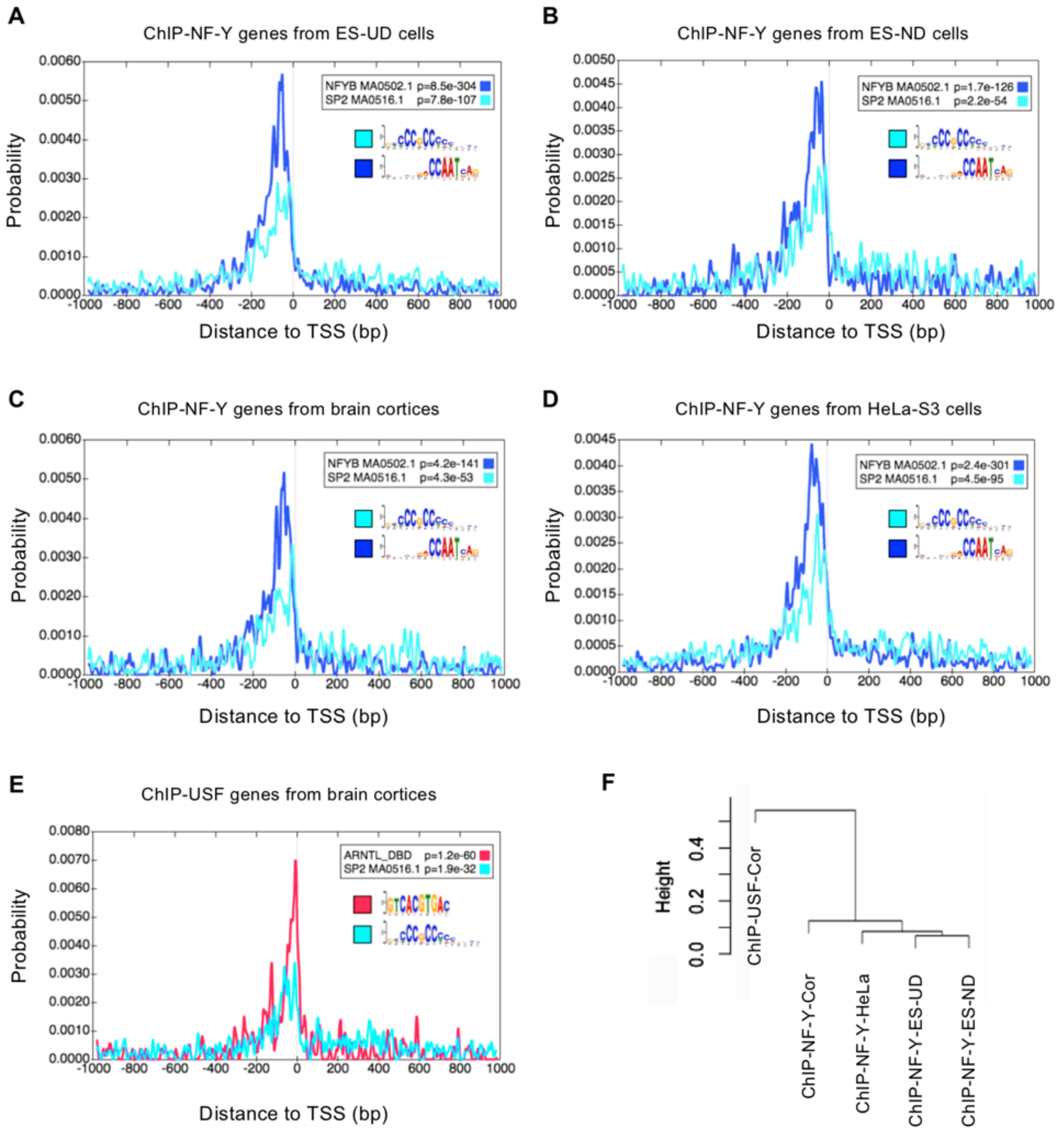

Supplementary Figure S6. Analysis of local enrichment of transcription factor binding motifs around TSSs of NF-Y-ChIP genes.

Motif enrichment analysis around TSSs was performed by CentriMo for top 500 of NF-Y-ChIP genes in mouse undifferentiated ES cells (ES-UD) (A), nuerally differentiated ES (ES-ND) (B), mouse brain cortices (C) and HeLa-S3 cells (D). (E) Motif enrichment analysis for USF1/2-ChIP genes in mouse brain cortices. (F) Hierarchical clustering of the cells / tissues based on the population of annotated ChIP genes (Ward.D2 method).

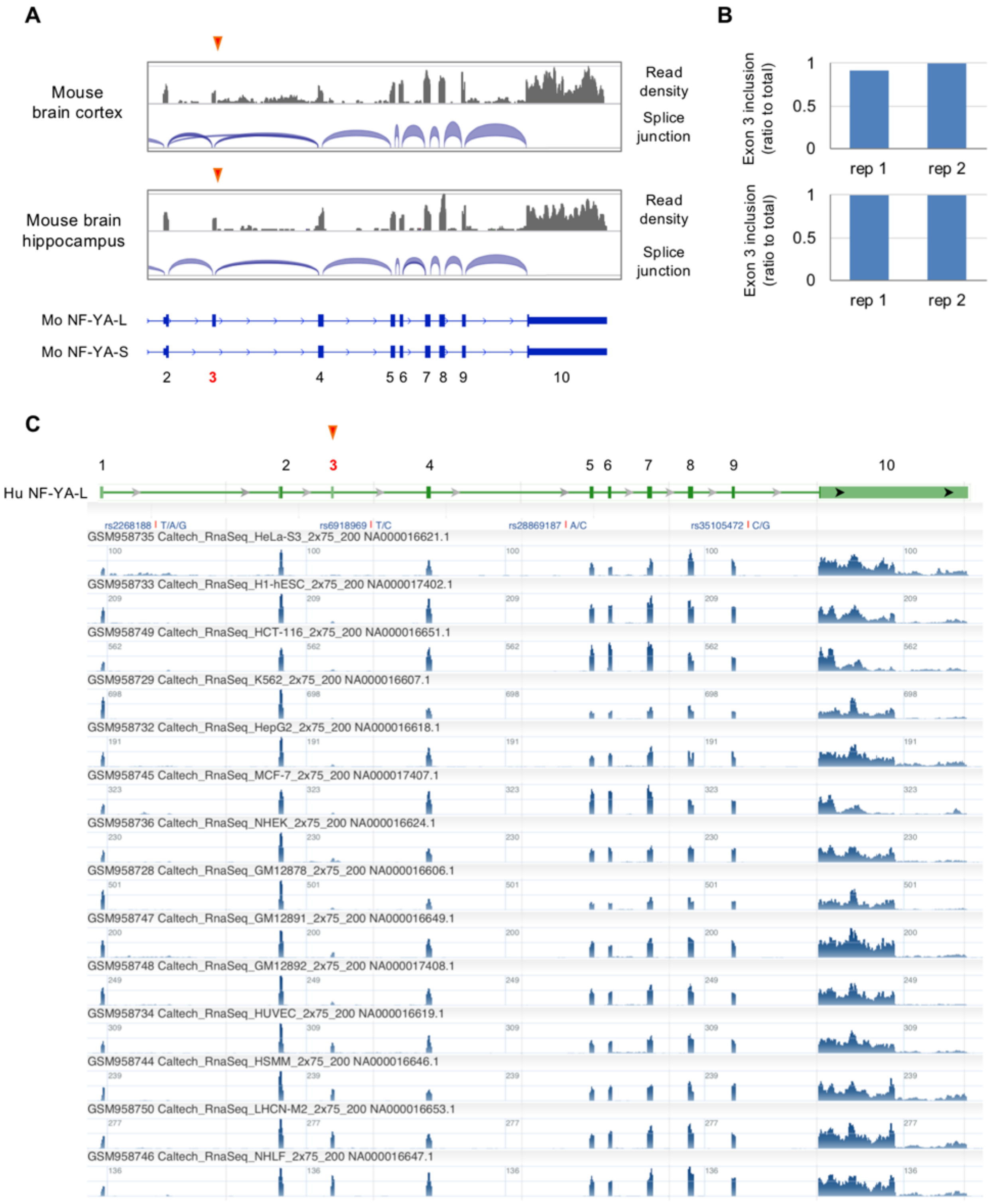

Supplementary Figure S7. NF-YA exon 3 expressions in mouse brain and human cell lines. (A) Integrative Genomics Viewer (IGV) shots of RNA-seq data showing read densities and splice junctions of NF-YA in mouse cortices and hippocampi. (B) Estimation of exon 3 inclusion (two replicates). (C) GEO genome viewer shot showing read densities of NF-YA in following human

cell lines; HeLa-S3 (cervical cancer), H1-hESC (human ES cells), HCT116 (colon cancer), K562 (myeloid leukemia), HepG2 (liver cancer), MCF7 (breast cancer), NHEK (epidermal keratinocyte), GM12878 (B-lymphocyte), GM12891 (B-lymphocyte), GM12892 (B-lymphocyte), HUVEC (umbilical vein endothelial cells), HSMM (skeletal myoblasts), LHCN-M2 (skeletal myoblasts), and NHLF (lung fibroblasts).

Figure 6E

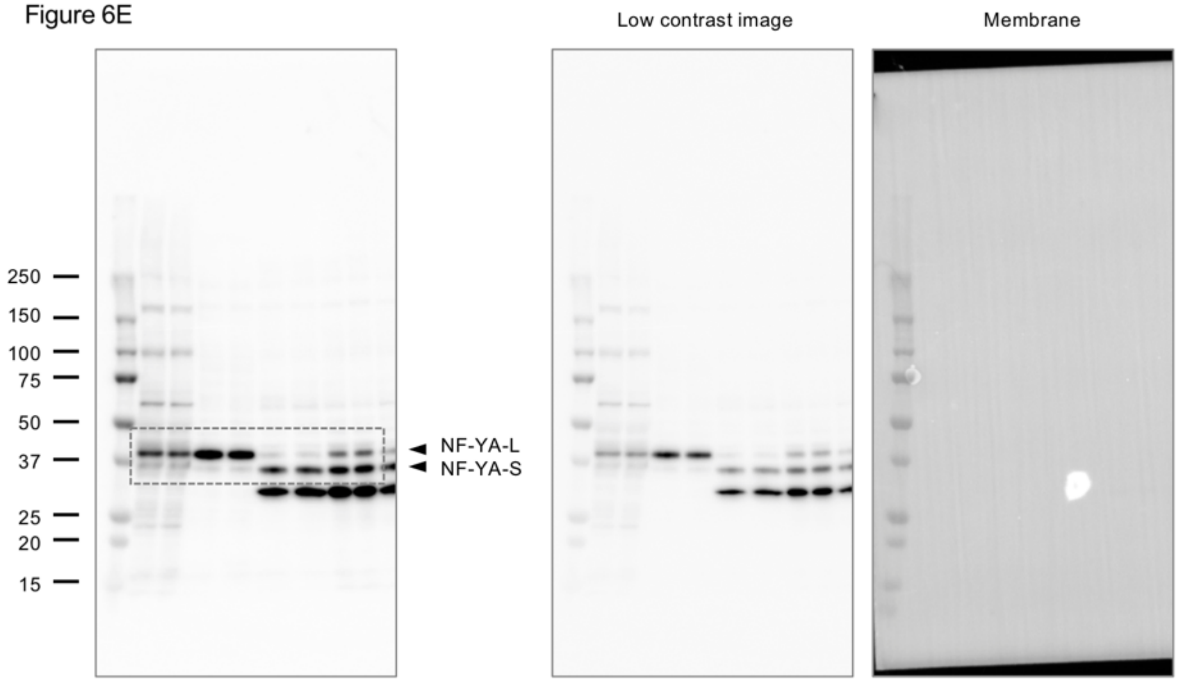

Figure S1A

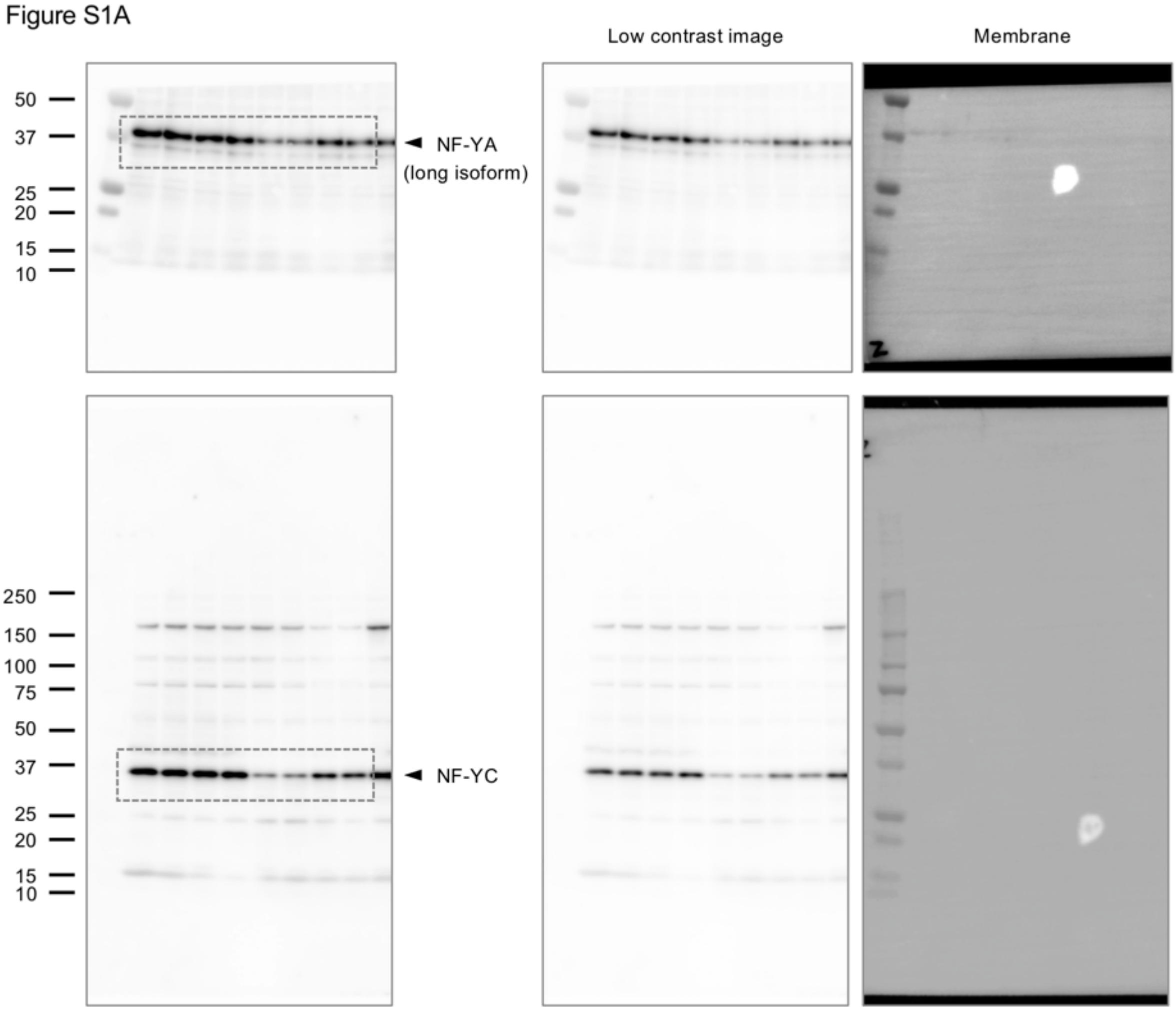

Figure S1A

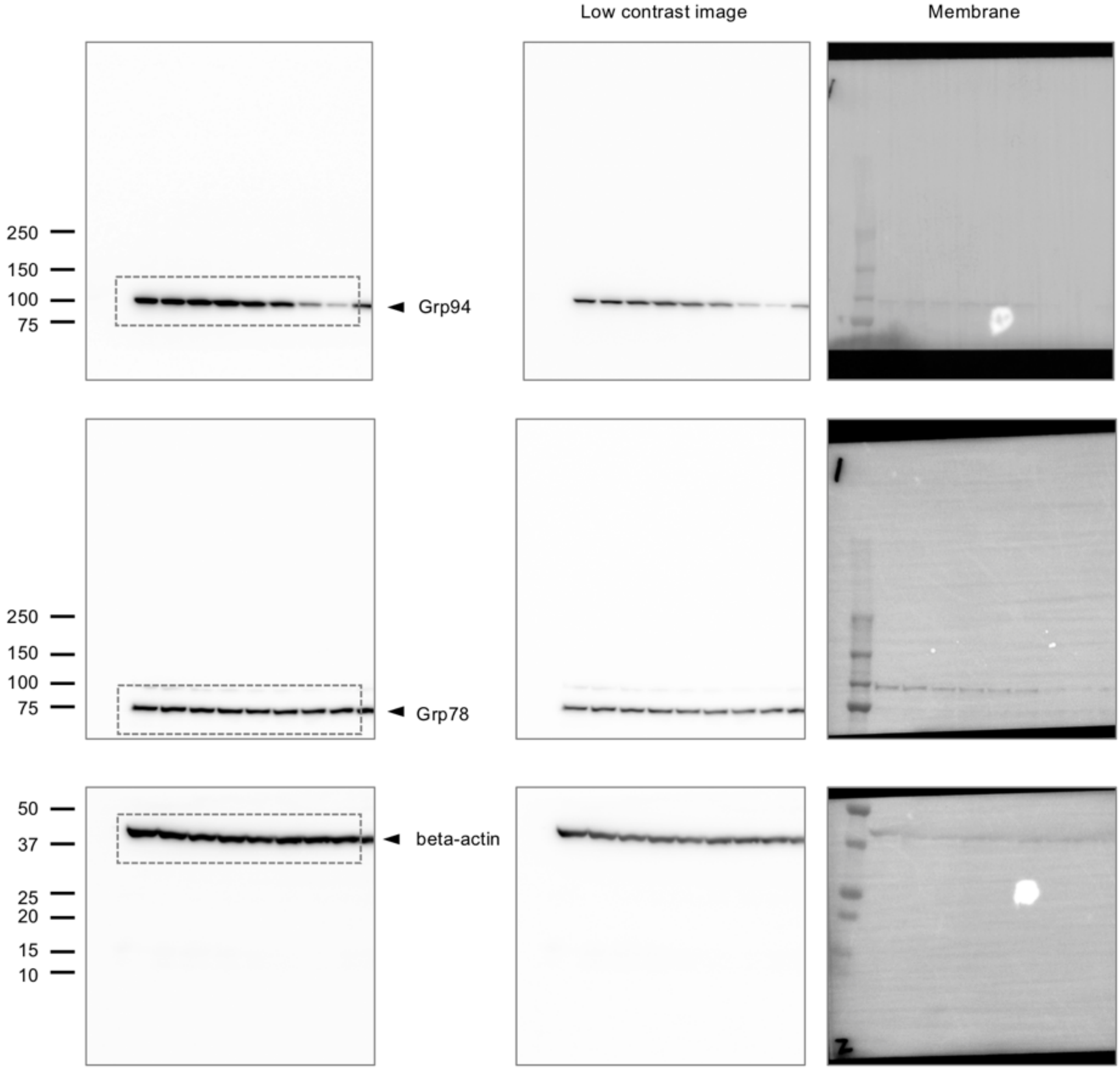

Supplementary Figure S8. Full-length images of Western blots.

Regions enclosed by dotted lines in left panels are shown in the figures. Lower contrast images are shown in middle panels and blot membrane images are shown in right panels. In some of the blots, blot membranes were cut horizontally to detect proteins with different molecular weights, such as Grp78 and beta-actin, at the same time.
